# Effects of Fertilizer on the Quality and Traceability of Tibet highland Barley (*Hordeum vulgare* L.): A Diagnosis Using Nutrients and Mineral Elements

**DOI:** 10.3390/foods11213397

**Published:** 2022-10-27

**Authors:** Shanshan Zhao, Cheng Qiu, Tangwei Zhang, Xiangyu Hu, Yan Zhao, Xiyu Cheng, Yuxuan Ma, Mengjie Qie, Chang Chen

**Affiliations:** 1 Institute of Quality Standard & Testing Technology for Agro-Products, Chinese Academy of Agricultural Sciences, Beijing 100081, China; 2 Institute of Agricultural Product Quality Standard and Testing Research, Tibet Academy of Agricultural and Animal Husbandry Sciences, Lhasa 850032, China; 3 College of Food Science and Engineering, Shandong Agricultural University, Taian 271018, China; 4 College of Life Sciences and Bioengineering, School of Science, Beijing Jiaotong University, Beijing 100044, China; 5 College of Food Science and Engineering, Inner Mongolia Agricultural University, Hohhot 010018, China

**Keywords:** highland barley, traceability, nutrients, mineral elements, chemometrics, fertilizer

## Abstract

Production areas influence the quality of highland barley (*Hordeum vulgare* L.), and fertilization levels may be associated with the origin traceability of highland barley. As the main object of the study, a collection of highland barley was planted in different areas in Tibet, China, to explore the effect of fertilizer on the quality and traceability of highland barley. We carried out field experiments with and without fertilizer treatment (using urea and diamine phosphate). Highland barley was distinguished by nutrient and mineral element contents in combination with chemometric methods. The results indicated that fertilizer treatment significantly affected some mineral element contents in highland barley and improved the accuracy of highland barley traceability. The combination of nutrients and mineral elements could distinguish highland barley from those raised in other areas due to influence of growing environment. P, K, Fe, and Cu provided a great contribution to the classification of highland barley. Thus, the combination of nutrients and mineral elements can be used as a powerful tool to track highland barley, indicating that fertilization treatment should be considered when tracing highland barley.

## 1. Introduction

Tibet is located in the southwest of the Qinghai Tibet Plateau, with an average altitude of more than 4000 m. Due to the unique topography, high altitude, and weather system of the Tibetan Plateau, a complex and diverse climate has been formed. Highland barley is mainly distributed in the naked barley area of the Qinghai Tibet Plateau. It has become the main food crop in the Plateau and important raw material for brewing, feed crops, and food because of its unique strong cold tolerance, wide adaptability, high yield, and early maturity [1,2]. Highland barley has high protein, high vitamin, high fiber, low sugar, and low fat, which is consistent with the “three high and two low” dietary structure [3]. The content of β-glucan, which plays a significant role in preventing cardiovascular diseases, diabetes, and so on, is high in highland barley [4]. In addition, highland barley is also rich in maternal fertility phenols, flavonoids, anthocyanins, and other phenolic substances [5,6,7]. These substances can be used as natural antioxidants and have unique physiological effects, such as anti-cancer, anti-aging, preventing cardiovascular diseases, and improving immunity [8,9], which has made highland barley gradually become a research hotspot in recent years.

Mineral element contents in the regional environment have their own characteristics. Plants absorb mineral elements from the environment in which they live. Therefore, mineral element analysis can be used as an effective technique for traceability and authenticity identification of plant origin [10,11,12,13]. A complex and diverse climate leads to typical fingerprint characteristics of mineral elements in highland barley [14]. It was reported that the producing area significantly affected the mineral element content of highland barley [15]. In our previous research, nutrient composition and mineral element contents of highland barley varied with origin [16]. Therefore, mineral element analysis is an effective means to distinguish highland barley-producing areas.

With the increasing requirements of modern consumers on the nutritional performance of food [17], improving the nutritional quality level is not only the focus of breeding, but also the focus of food. Fertilizers are extremely important factors, and directly determine plant yield, quality, and nutritional content as well as the composition and content of essential trace elements [18,19]. They are one of the material bases of agricultural production. The improvement of plant nutrition and mineral elements through reasonable fertilizer treatment is of great significance to the improvement of plant yield and quality. Fertilizers may change the mineral element contents in agricultural products [20]. With treatment using fertilizers, the iron (Fe), zinc (Zn), copper (Cu), and manganese (Mn) concentrations increased in rice, which indicated that transport ability of trace elements was enhanced after nitrogen (N) application [21]. It is also reported that fertilizer increased the concentration and accumulation of Cu and Zn in wheat [22]. While the effect of fertilizers on the elemental content of produce has been studied, their effect on traceability was seldom reported. The natural farming environment in the Qinghai Tibet Plateau area is obviously different compared to the plain areas. However, there is no research on whether fertilizer can improve the nutrition and mineral elements of the plants in the Qinghai Tibet Plateau area. Revealing the influence of fertilizer on the quality and traceability of Tibet highland barley will not only provide reference for the planting of plateau crops, but also provide data support for tracing the origin of highland barley.

In this study, a collection of highland barley planted in different areas of Tibet was investigated. The field experiment included two groups: fertilizer treatment and no fertilizer treatment. The content of mineral elements and nutrients in highland barley was determined after it was mature. The purposes of the study were: (i) to investigate the effect of fertilizer treatment on the content of the mineral elements and nutrients in highland barley; (ii) to study the influence of fertilizer treatment on the traceability of production areas; (iii) to determine the indicators that have an important effect on the traceability of highland barley samples.

## 2. Materials and methods

### 2.1. Sampling Information

Highland barley seeds were selected from the same source and same type and then planted in five areas (Lhasa, Nyingchi, Ngari, Shannan, and Xigaze) in Tibet. Highland barley was planted under the same ecological condition in each area with/without nitrogenous fertilizer treatment (urea and diamine phosphate). After the highland barley had matured, 70 highland barley samples were collected from these areas, including Lhasa (n = 11), Nyingchi (n = 13), Ngari (n = 15), Shannan (n = 16), and Xigaze (n = 15). Table 1 shows the collection information of highland barley.

### 2.2. Preparation of Samples

The highland barley sample was washed with ultrapure water and air-dried until completely dry. These samples were then ground and sieved through a 100-mesh sieve (<150 microns).

### 2.3. Determination of Nutrients

#### 2.3.1. Starch Content

According to the national standard determination of starch in food by acid hydrolysis method (GB/T 5009.9-2008), after fat and soluble sugar were removed from the samples, the starch was hydrolyzed into reducing sugar by acid, after which the reducing sugar content was calculated and converted into starch content.

#### 2.3.2. Crude Protein Content

The protein content in highland barley was determined using the Kjeldahl method, according to the national standard Kjeldahl method for determination of protein in food (GB 5009.5-2010). The proteins in highland barley were broken down by catalytic heating. Ammonium sulfate was formed by the combination of sulfuric acid and ammonia. Ammonia was released and then absorbed by boric acid after alkalization distillation. The content of the released ammonia could be determined by titration of acid standard solutions. The protein content was then calculated by multiplying the amount of acid consumed by the conversion factor.

#### 2.3.3. Crude Fiber Content

The crude fiber content was determined according to the national standard method for determination of crude fiber in plant foods (GB/T 5009.10-2003). Boiling sulfuric acid was added to the highland barley, then heated to be slightly boiling, keeping the volume constant, maintained for 30 min, with sugar, pectin, starch, and hemicellulose in highland barley hydrolysis. After filtration, the solution was washed with boiling water until it was not acidic. Then, the residue was washed into a conical flask with boiled potassium hydroxide solution and heated for 30 min. After that, the residue was filtered immediately and then washed with boiling water 2~3 times to remove proteins and fatty acids. It was then dried and weighed until constant and the remaining residue was crude fiber.

#### 2.3.4. β-Glucan Contents

The contents of β-glucan were analyzed by spectrophotometry according to the determination of p-glucan in cereal and its products (NY/T 2006–2011). Lichen glyanase specifically hydrolyzed β-glucan to oligosaccharide. β-glucosidase hydrolyzed oligosaccharides into glucose. Glucose in the action of glucose oxidase produced gluconic acid and hydrogen peroxide. Under the action of peroxidase, hydrogen peroxide was oxidized and condensed with 4-aminoantipyrine to form red quinones. The red quinones absorbance was at 510 nm, and its absorbance value was proportional to the glucose content.

#### 2.3.5. Total Flavonoid Contents

The contents of total flavonoids were analyzed by sodium nitrite aluminum chloride complex spectrophotometry according to the determination of flavones in sophora japonica and its products’ spectrophotometric method (DB 34/T 2743-2016). In the presence of neutral or weakly alkaline sodium nitrite, flavonoids formed chelates with aluminum salts. After adding NaOH, the solution turned orange. In a certain concentration range, its absorbance was in direct proportion to the content of flavonoids. It conformed to the Lambert–Beer law.

### 2.4. Element Analysis

#### 2.4.1. Phosphorus Content

The phosphorus content in highland barley was determined as follows: after digestion of highland barley, ammonium phosphomolybdate was formed by the combination of phosphorus and ammonium molybdate under acidic conditions. Ammonium phosphomolybdate was then reduced to blue compound molybdenum blue with hydroquinone, sodium sulfite, or stannous chloride and hydrazine sulfate. The concentration of phosphorus was determined by the absorbance value of molybdenum blue at 660 nm against the analytical curve. 

#### 2.4.2. Contents of Other Mineral Elements

A 1.00 g sample was weighed into a high-pressure digestion tank, to which 10 mL of mixed acid (V_nitric acid_: V_perchloric acid_, 9:1) was added. The mixture was heated and digested on an anti-corrosion electric heating plate (JKHF-140L, Qingdao Zico Experimental Instrument Co. Ltd., China) for 1 h at 120 °C. Then, temperature was raised to 150 °C, when smoke appeared, and the temperature was increased to 180 °C until the solution was transparent. The precipitate was diluted to 50 mL using 1% HNO_3_ solution. Finally, samples were filtered through a 0.45 μm syringe filter for further analysis. Cu, Ca, Zn, K, Mn, K, Fe, and Mg were determined by Inductively Coupled Plasma-Optical Emission Spectrometry (ICPOES, Varian 710, Agilent, Santa Clara, CA, USA). GBW 10046 (Institute of Geophysical and Geochemical Exploration, Chinese Academy of Geological Sciences) was used for calibration verification as a matrix reference material. 

Measurement conditions: ICP: RF power: 1150 W, pump speed: 50 rpm, auxiliary gas flow: 0.5 mL/min, atomizer gas flow: 0.55 mL/min.

### 2.5. Statistical Analysis

SPSS 22.0 (International Business Machines Corporation, Armonk, NY, USA) was used for statistical analysis. Significant differences in the contents of nutrients and mineral elements between the unfertilized and the fertilized highland barley were determined by Student’s *t*-test (*t*-test). A post hoc Duncan’s multiple comparison was performed to determine the significance of differences between the samples from different areas in one-way analysis of variance (ANOVA). Linear discriminant analysis (LDA) was used to evaluate the classification of samples from different sources. SIMCA 14.1 software (Umetrics, Umea, Sweden) was used for principal component analysis (PCA), partial least squares-discriminant analysis (PLS-DA), and hierarchical cluster analysis (HCA) in order to cluster and distinguish the samples from different areas. Variable importance projection (VIP) list was obtained from these analyses.

## 3. Results and Discussion

### 3.1. Differences in the Nutrient and Mineral Element Contents between Unfertilized and Fertilized Highland Barley Samples

The contents of five nutrients and eight elements in the fertilized and the unfertilized highland barley samples are shown in Table 2. *t*-test results demonstrated the contents of crude protein, total flavonoids, Cu, Fe, and Zn in the unfertilized and the fertilized highland barley from all areas had no significant difference (*p* > 0.05). When the nitrogen in the fertilizer enters the soil, it may have an antagonistic or synergistic effect on other trace elements and influence the trace element contents in the soil, so not all the elements in plants will increase [22]. Jiang et al. found that the application of nitrogen fertilizer increased the content and accumulation of Zn and Cu in wheat, while it inhibited the content and accumulation of Mn in wheat [22]. The contents of different trace elements differ in their reaction to nitrogen fertilizer. On the other hand, nitrogen fertilizer will change the pH of soil, and a change in soil PH affects the activity of trace elements and the solubility of compounds in soil, thus affecting the absorption of crops. When soil pH < 5.5, Cu and Zn could be better absorbed by crops, while when pH > 6.5, the ability of these elements to enter the crop was significantly reduced [23]. 

The Ca, K, and Mn contents in the fertilized highland barley from Shannan and the Ca, Mg, and Mn contents in the fertilized highland barley from Xigaze were significantly higher than those in their corresponding unfertilized highland barley. The contents of starch, crude fiber, and Ca in the fertilized highland barley from Nyingchi and the contents of β-glucan, Ca, K, and Mn in the fertilized highland barley from Lhasa were significantly lower than those in their corresponding unfertilized highland barley. This may be a result of the high altitude of Shannan and Xigaze, which belong to an alpine climate, while Nyingchi and Lhasa belong to a valley climate. A reasonable speculation is that climate change may cause changes in soil physicochemical properties or directly affect plant uptake of trace elements in fertilizers. After fertilization, an alpine climate promotes the absorption of mineral elements by highland barley, and a valley climate inhibits the absorption of mineral elements by highland barley. It is believed that any change in environmental factors will affect the absorption of mineral elements by plants [24].

There was no significant difference in 12 out of the 13 indexes between the fertilized highland barley and the unfertilized highland barley from Ngari, in which only the content of β-glucan was significantly reduced. This may be a result of the lower temperature in Ngari. The lower temperature reduced the absorption rate of mineral elements by the crop root from fertilizer. A decrease in temperature changes the structural characteristics of plant cell membranes, resulting in a decrease in cell membrane fluidity [25], and it is difficult for metal ions to be passively and actively transported into plants through the cell membrane. A decrease in temperature also inhibits metabolism, reduces the metal binding sites on the cell membrane, and leads to fewer released compounds conducive to the absorption of metal ions from the fertilizer [25,26]. It can be seen that the use of fertilizer does not necessarily increase nutrient and mineral element contents, because the absorption of nutrients and mineral elements by highland barley from the fertilizer is mainly dominated by the environmental factors and the fertilizer itself.

### 3.2. Differences in Nutrient and Mineral Element Contents in Highland Barley Samples from Different Regions

Some nutrient contents were different (*p* < 0.05) in the highland barley samples from different regions according to post hoc Duncan’s testing of ANOVA testing (Table 2). The contents of starch (Unfertilized: 46.36 ± 1.79%; Fertilized: 45.34 ± 1.59%) in the highland barley samples from Shannan were the lowest. The contents of crude protein (Unfertilized: 7.59 ± 1.61%; Fertilized: 8.72 ± 1.70%) in the highland barley samples from Ngari were lower than those from the other four areas. Crude fiber contents (Unfertilized: 4.11 ± 0.22%; Fertilized: 3.58 ± 0.40%) in Nyingchi highland barley samples were the highest. These results showed that different ecological conditions will have significant impact on the nutritional quality of highland barley, which is consistent with previous conclusions [27]. The β-glucan contents ranged from 4.02% to 5.60%, which was basically consistent with the previous report [28]. The total flavonoid contents in Ngari highland barley were the highest (Unfertilized: 0.27 ± 0.08%; Fertilized: 0.25 ± 0.07%), while those in Nyingchi highland barley were the lowest (Unfertilized: 0.18 ± 0.02%; Fertilized: 0.19 ± 0.02%). Flavonoid plays a key role in the resistance of plants to the pressure caused by the external environment [29]. The altitude of Ngari area is up to 3900 m, and the annual rainfall is only 172.8 mm. This extreme environment is conducive to the accumulation of total flavonoids in highland barley. Nyingchi belongs to the valley plateau area, and the climate is warm and humid, so highland barley contains the lowest contents of total flavonoids. 

The contents of some mineral elements were different (*p* < 0.05) in highland barley samples from different regions based on ANOVA using a post hoc Duncan’s test (Table 2). Due to the effect of growing environment on the composition of highland barley, mineral element contents in highland barley were strongly related to regions. Each region had a characteristic element content fingerprint. The element content in plants’ environment (including soil and air) has great influence on the composition of plant [30]. Ca, Cu, K, Mg, Mn, and Zn contents in the highland barley samples from Lhasa and Xigaze were similar, which may result from the fact that Lhasa is adjacent to Xigaze, and these two areas have similar latitude, longitude, and geological conditions, as well as being equally rich in minerals [31]. Compared with the other areas, the highland barley samples from Shannan contained the lowest contents of K (Unfertilized: 2110.28 ± 175.98 mg/kg; Fertilized: 2534.69 ± 282.32 mg/kg) and Ca (Unfertilized: 17.93 ± 8.27 mg/kg; Fertilized: 35.07 ± 10.63 mg/kg). The altitude of Shannan area is up to 4000 m, and the annual rainfall is 293.1 mm. Under high altitude and low annual rainfall, highland barley suffered from water stress. Water stress on highland barley guard cells resulted in a large loss of potassium and calcium ions in highland barley, leading to significantly decreased contents of K and Ca [32]. Zheng et al. studied the influence of water stress on the mineral ion content in rape seedlings and proved that the contents of potassium and calcium decreased significantly [33]. 

The contents of Cu (Unfertilized: 59.25 ± 10.47 mg/kg; Fertilized: 59.44 ± 11.03 mg/kg) in the highland barley samples from Ngari were the highest due to high altitude. Ngari has a cold and dry climate, with annual rainfall of 172.8 mm. It has been found that, with a decrease in temperature, the content of Cu in plants increased [34]. The contents of Cu (Unfertilized: 3.20 ± 0.39 mg/kg; Fertilized: 3.25 ± 0.69 mg/kg) in Nyingchi highland barley were the lowest. The climate in Nyingchi area is completely opposite to that in other areas. Nyingchi’s climate type is tropical humid and sub-humid, and the annual rainfall is 977 mm. With a warm climate and sufficient precipitation, the Cu contents in Nyingchi highland barley were the lowest. The Ca, Mg, and Mn contents in Xigaze highland barley, the Fe content in Shannan highland barley, and the Zn content in Shannan and Ngari highland barley were the highest, due to the higher altitude of these areas. With an increase in altitude, CO_2_ concentration in the air decreases, and the absorption of large and trace elements by plants increases [35,36]. In general, due to the influence of environmental factors, nutrient and mineral element contents in highland barley from different areas varied. 

### 3.3. Chemometric Analysis of Highland Barley Samples from Different Regions

For unfertilized highland barley, the accuracy of original classification and cross-validation were 100% and 87.0%, respectively. For fertilized highland barley, the accuracy of original classification and cross-validation were 100% and 91.7%, respectively, in which the cross-validation rate increased by 4.7% (Table 3) because the absorption of mineral elements by highland barley from the fertilizer is influenced by environmental conditions. After fertilization, the alpine climate promotes the absorption of mineral elements by highland barley, and the valley climate inhibits the absorption of mineral elements by highland barley. Therefore, some mineral element contents in the highland barley samples from different producing areas were significantly different after fertilization. Discrimination accuracy has been improved. The combination of nutrients and mineral elements can be used to trace highland barley. This was consistent with our previous results [16].

**Figure 1:**
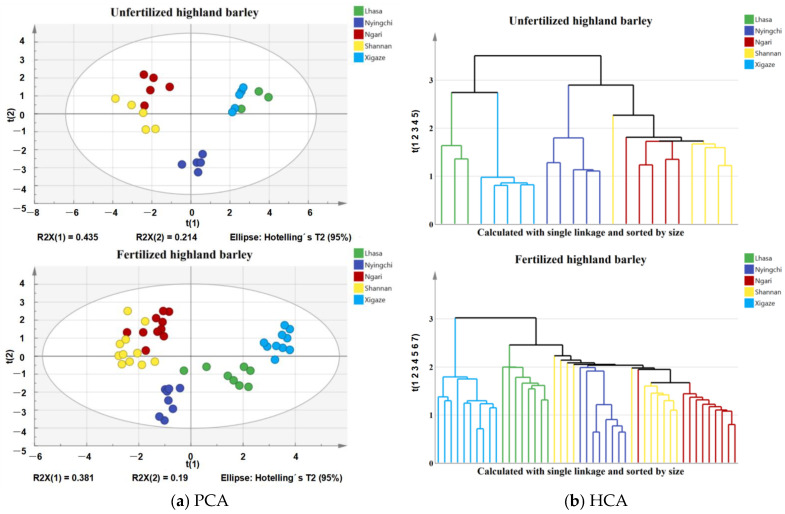
The PCA and HCA of fertilized and unfertilized highland barley samples from five regions of Tibet with data on nutrients and mineral elements: (**a**) PCA; (**b**) HCA.

Nutrient and mineral element contents in fertilized and the unfertilized highland barley were analyzed by PCA and HCA (Figure 1). In the PCA score plots, the first two PCs contained most of the valid information used for classification. For unfertilized highland barley, the first two PCs (i.e., PC1 and PC2) contributed 0.435 and 0.214 of the variances, respectively. For fertilized highland barley, the first two PCs (i.e., PC1 and PC2) contributed 0.381 and 0.19 of the variances, respectively. Highland barley from different regions can be clearly classified based on nutrient and mineral element contents. The highland barley samples from Ngari and Shannan overlap due to the similar climatic conditions of both areas [30]. The results obtained by HCA are shown as a tree view in which five clear clusters are visible. The clustering behavior of the highland barley samples appears to differ between HCA and PCA. This may simply result from the use of PC1 and PC2. In conclusion, both PCA and HCA can classify highland barley. PCA and HCA showed the classification information of samples by the position of each sample point on the score map without prior classification, so the classification results are more objective [37]. HCA captures similarity more effectively than PCA from a visual perspective [38]. Compared with the unfertilized highland barley, the classification in the PCA score plots of samples after fertilization from different regions was more obvious. This was consistent with the classification results of highland barley by LDA because of the environmental conditions.

**Figure 2:**
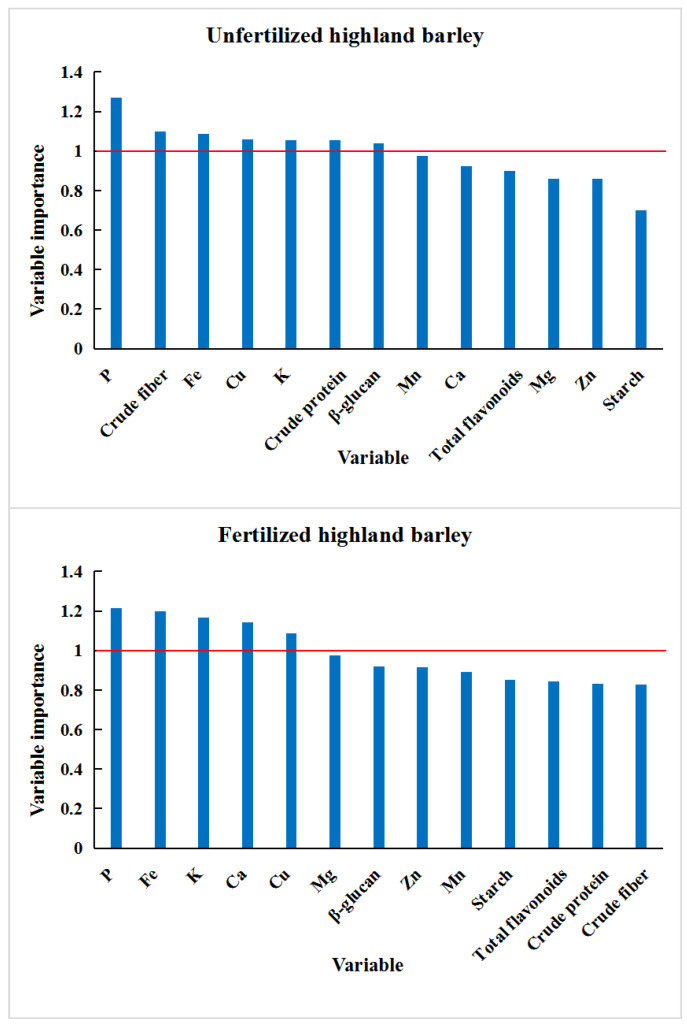
Variable importance projection of fertilized and unfertilized highland barley samples from five regions of Tibet with data on nutrients and mineral elements.

The VIP value (Figure 2) can quantify the contribution of each variable to the classification. The larger the VIP value, the more significant the difference in the variable among different regions of highland barley [37]. For the unfertilized highland barley, seven variables (P, crude fiber, Fe, Cu, K, crude protein, and β-glucan) have higher importance values (>1). For the fertilized highland barley, only five variables (P, Fe, K, Ca, and Cu) have higher importance values (>1). This may be a result of the fact that the differences in the nutrient and mineral element contents in highland barley from different areas become larger after fertilization. Fewer indicators can distinguish highland barley samples from different areas. Common variables included P, K, Fe, and Cu. These indicators were highly correlated with the producing area of highland barley and can provide certain theoretical support for the traceability of the producing area of highland barley.

## 4. Conclusions

In this research, highland barley was used as the material and planted in five areas in Tibet. Highland barley from five regions of Tibet was identified by chemometric methods combined with the analysis of nutrient composition and mineral elements. We found that even if these five areas belonged to the Qinghai Tibet Plateau, they varied significantly in altitude, precipitation, and temperature. Nutrient and mineral element contents in highland barley samples from different areas were different due to the influence of growing environment. Thus, the combination of nutrient and mineral element contents can be used as a powerful tool to track highland barley. P, K, Fe, and Cu provided a great contribution to the classification of highland barley for traceability.

Due to the antagonistic or synergistic effect of nitrogen in fertilizer on other elements and the influence of environmental factors, there are significant differences in the contents of some nutritional and mineral elements between the fertilized highland barley and the unfertilized highland barley. After fertilization, environmental factors promoted/inhibited the absorption of mineral elements by highland barley and improved the accuracy of highland barley traceability. Therefore, fertilization treatment should be considered when tracing highland barley.

## Figures and Tables

**Table 1:** Information on highland barley samples from five regions of Tibet.

Region	Fertilized or Not	Number	Altitude (m)	East Longitude	North Latitude	Yearly Rainfall (mm)	Yearly Temperature (°C)
Lhasa	Unfertilized	3	3600	90.90	29.38	444.8	7.5
	Fertilized	8					
Nyingchi	Unfertilized	5	2700	95.56	30.28	977	8.5
	Fertilized	8					
Ngari	Unfertilized	5	3900	81.17	30.29	172.8	3
	Fertilized	10					
Shannan	Unfertilized	5	4000	91.88	28.97	293.1	8.2
	Fertilized	11					
Xigaze	Unfertilized	5	4006	89.56	28.94	304.3	5
	Fertilized	10					

**Table 2:** Values of nutrients and mineral elements in highland barley from different regions.

Index	Fertilized or Not	Lhasa	Nyingchi	Ngari	Shannan	Xigaze
Starch (%)	Unfertilized	49.86 ± 0.96 ^b^	48.59 ± 2.35 ^ab^	46.74 ± 1.56 ^a^	46.36 ± 1.79 ^a^	49.82 ± 0.84 ^b^
	Fertilized	48.76 ± 1.65 ^b^	45.73 ± 1.69 ^a^	46.29 ± 1.65 ^a^	45.34 ± 1.59 ^a^	48.78 ± 1.01 ^b^
	*p*-Value	0.31	0.02	0.62	0.27	0.08
Crude protein (%)	Unfertilized	10.58 ± 1.37 ^b^	9.90 ± 2.95 ^ab^	7.59 ± 1.61 ^a^	10.21 ± 0.84 ^ab^	8.63 ± 0.99 ^ab^
	Fertilized	9.88 ± 2.80 ^ab^	11.55 ± 1.79 ^b^	8.72 ± 1.70 ^a^	11.01 ± 2.32 ^b^	9.60 ± 1.04 ^ab^
	*p*-Value	0.70	0.32	0.24	0.33	0.13
Crude fiber (%)	Unfertilized	2.80 ± 0.35 ^a^	4.11 ± 0.22 ^d^	3.17 ± 0.30 ^b^	3.69 ± 0.12 ^c^	3.27 ± 0.22 ^b^
	Fertilized	3.39 ± 0.51 ^bc^	3.58 ± 0.40 ^c^	2.92 ± 0.55 ^a^	3.54 ± 0.30 ^c^	3.12 ± 0.29 ^ab^
	*p*-Value	0.10	0.03	0.36	0.20	0.39
β-glucan (%)	Unfertilized	5.51 ± 0.09 ^c^	4.61 ± 0.43 ^ab^	5.27 ± 0.35 ^bc^	5.01 ± 0.78 ^abc^	4.49 ± 0.31 ^a^
	Fertilized	4.43 ± 0.41 ^a^	4.60 ± 0.29 ^ab^	4.73 ± 0.34 ^abc^	5.04 ± 0.38 ^c^	4.84 ± 0.38 ^bc^
	*p*-Value	0	0.79	0.01	0.92	0.05
Total flavonoids (%)	Unfertilized	0.20 ± 0.03	0.18 ± 0.02	0.27 ± 0.08	0.22 ± 0.06	0.27 ± 0.08
	Fertilized	0.22 ± 0.05 ^ab^	0.19 ± 0.02 ^a^	0.25 ± 0.07 ^b^	0.21 ± 0.04 ^a^	0.23 ± 0.04 ^ab^
	*p*-Value	0.58	0.25	0.73	0.48	0.21
P (mg/kg)	Unfertilized	4320.79 ± 206.71 ^b^	4957.91 ± 351.29 ^c^	3713.41 ± 526.56 ^a^	3729.91 ± 138.73 ^a^	3328.63 ± 348.55 ^a^
	Fertilized	4113.58 ± 359.54 ^c^	5350.46 ± 433.62 ^d^	3592.10 ± 371.05 ^b^	3953.70 ± 567.47 ^bc^	3171.35 ± 275.91 ^a^
	*p*-Value	0.38	0.05	0.61	0.41	0.21
Ca (mg/kg)	Unfertilized	471.63 ± 104.42 ^c^	41.59 ± 11.68 ^ab^	90.84 ± 34.36 ^b^	17.93 ± 8.27 ^a^	425.81 ± 50.36 ^c^
	Fertilized	101.70 ± 55.05 ^c^	18.79 ± 9.61 ^a^	89.71 ± 29.31 ^bc^	35.07 ± 10.63 ^ab^	595.75 ± 125.75 ^d^
	*p*-Value	0	0	0.95	0.01	0
Cu (mg/kg)	Unfertilized	3.66 ± 0.59 ^a^	3.20 ± 0.39 ^a^	59.25 ± 10.47 ^c^	38.78 ± 21.12 ^b^	5.97 ± 0.75 ^a^
	Fertilized	3.54 ± 0.42 ^a^	3.25 ± 0.69 ^a^	59.44 ± 11.03 ^c^	40.81 ± 10.83 ^b^	5.79 ± 1.26 ^a^
	*p*-Value	0.71	0.67	0.97	0.80	0.78
Fe (mg/kg)	Unfertilized	130.75 ± 22.81 ^abc^	64.82 ± 12.48 ^a^	154.02 ± 53.09 ^bc^	196.84 ± 76.02 ^c^	86.57 ± 26.91 ^ab^
	Fertilized	178.60 ± 54.47 ^bc^	67.81 ± 17.74 ^a^	123.38 ± 32.25 ^ab^	208.76 ± 134.25 ^c^	130.15 ± 66.68 ^ab^
	*p*-Value	0.19	0.47	0.18	0.86	0.06
K (mg/kg)	Unfertilized	8631.61 ± 293.48 ^d^	5069.77 ± 524.57 ^c^	3711.78 ± 393.96 ^b^	2110.28 ± 175.98 ^a^	5343.72 ± 324.24 ^c^
	Fertilized	6107.49 ± 636.79 ^e^	4458.54 ± 447.79 ^c^	3633.97 ± 408.66 ^b^	2534.69 ± 282.32 ^a^	5456.81 ± 388.93 ^d^
	*p*-Value	0	0.10	0.73	0.01	0.60
Mg (mg/kg)	Unfertilized	990.28 ± 212.95 ^c^	391.12 ± 43.59 ^b^	218.25 ± 14.07 ^a^	157.45 ± 19.79 ^a^	896.54 ± 39.05 ^c^
	Fertilized	495.02 ± 51.02 ^c^	309.03 ± 65.62 ^b^	216.62 ± 39.03 ^a^	183.46 ± 28.48 ^a^	979.50 ± 48.02 ^d^
	*p*-Value	0.05	0.07	0.91	0.09	0
Mn (mg/kg)	Unfertilized	10.82 ± 1.07 ^c^	4.33 ± 0.95 ^b^	4.87 ± 1.06 ^b^	2.03 ± 0.10 ^a^	10.00 ± 1.34 ^c^
	Fertilized	7.94 ± 1.09 ^c^	3.84 ± 0.65 ^ab^	4.56 ± 1.73 ^b^	2.90 ± 0.75 ^a^	12.41 ± 1.80 ^d^
	*p*-Value	0	0.27	0.73	0.02	0
Zn (mg/kg)	Unfertilized	26.23 ± 1.92 ^a^	28.80 ± 4.14 ^a^	52.22 ± 6.25 ^b^	48.08 ± 7.50 ^b^	29.20 ± 2.96 ^a^
	Fertilized	25.06 ± 7.56 ^a^	32.23 ± 4.62 ^b^	46.69 ± 8.89 ^c^	44.03 ± 7.47 ^c^	27.53 ± 3.60 ^ab^
	*p*-Value	0.80	0.15	0.24	0.33	0.32

Values are means ± SD. Numbers with different superscript are significantly (*p* < 0.05) different with respect to rows for the different areas: post hoc Duncan’s test.

**Table 3:** Classification of highland barley samples based on nutrients and mineral elements values by LDA.

Samples	Region	Lhasa	Nyingchi	Ngari	Shannan	Xigaze	Total
Unfertilized highland barley	Original classification (%)	100	100	100	100	100	100
	Cross-validation (%)	66.7	100	100	80	80	87.0
Fertilized highland barley	Original classification (%)	100	100	100	100	100	100
	Cross-validation (%)	100	100	80	81.8	100	91.7

## Data Availability

The data presented in this study are available on request from the corresponding author. The data are not publicly available due to the incomplete entire project.
